# Erratum for Alveolar Type II Cells Escape Stress Failure Caused By Tonic Stretch Through Transient Focal Adhesion Disassembly

**DOI:** 10.7150/ijbs.48633

**Published:** 2020-06-11

**Authors:** Xiao-Yang Liu, Xiao-Fei Chen, Yan-Hong Ren, Qing-Yuan Zhan, Chen Wang, Chun Yang

**Affiliations:** 1 Beijing Chao-Yang Hospital, Capital Medical University, China; 2 Beijing Institute of Respiratory Medicine, China; 3 Institute of Biomechanics and Medical Engineering, School of Aerospace, Tsinghua University, China

In our paper 1, the second panel of Figure 1c was mis-pasted. Fig. 1c should be corrected as follows.

All the authors have read the erratum and agree with the correction.

## Figures and Tables

**Figure 1:**
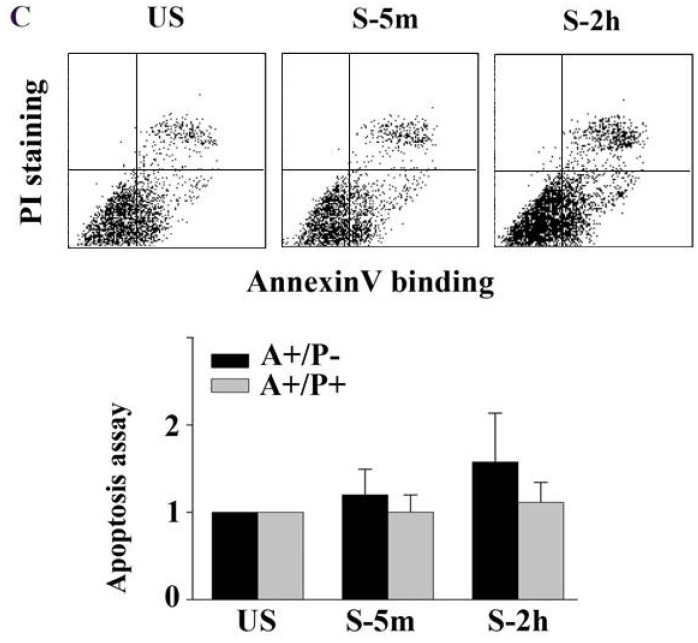
(A) Tonic stretch-induced ATII cell death over time (1 min, 5 min, 2 h, and 4h). Cytoplasm of viable cells was stained with calcein-AM (green), and nuclei of nonviable cells were stained with ethidium homodimer-1 (red). Bar = 200 μm. (B) Statistical analysis of stretch-induced cell death over time, the cell death rate did not increase after 5 min. Values were means ± SD (n=8). *P < 0.001 vs. unstretched cells (US); **P < 0.001 vs. S-1m. (C) Cells were stained with annexin V and PI, and analyzed by flow cytometry. AnnexinV-positive/PI-negative (lower right quadrant) represented the early apoptosis, annexinV-positive/PI-positive (upper right quadrant) represented the late apoptosis/necrosis. Apoptosis rate was not sig-nificantly different between US and stretch group (S-5m and S-2h). Values were means ± SD (n=8). (D) Tonic stretch-induced cell morphological remodeling was detected by SEM. During the stretch, cells experienced expansion (1 min), contraction (5 min), and reexpansion (2 h). Bar = 20 μm. (E) Stretch induced cell morphological remodeling. Im-munofluorescence of representative cells at the time indicated for actin (red), unclear (blue), and FA marker VIN (green). Bar = 10 μm. (F) Quantitative analysis of FAs fluorescence intensity during the stretch showed that cells experienced rapid FAs disassembly and sequential reassembly. * P < 0.01 vs. US; ** P < 0.01 vs. S-5m. Values were means ± SD (n>60).
